# Supplementary material for: Cancer-testis Antigen FATE1 Expression in Adrenocortical Tumors Is Associated with A Pervasive Autoimmune Response and Is A Marker of Malignancy in Adult, but Not Children, ACC
Source: Cancers (Basel). 2020 Mar 14;12(3):689. doi: 10.3390/cancers12030689 (PMC7140037; doi:10.3390/cancers12030689)
Supplement: Supplementary file 1 [file cancers-12-00689-s001.zip › cancers-719269-suppl/cancers-719269-supplementary-proofreading.docx]

Supplementary Materials

Cancer-testis Antigen FATE1 Expression in Adrenocortical Tumors Is Associated with A Pervasive Autoimmune Response and Is A Marker of Malignancy in Adult, but Not Children, ACC

Mabrouka Doghman-Bouguerra, Pascal Finetti, Nelly Durand, Ivy Zortéa S. Parise ,
Silviu Sbiera, Giulia Cantini, Letizia Canu, Ségolène Hescot, Mirna M.O. Figueiredo,
Heloisa Komechen, Iuliu Sbiera, Gabriella Nesi, Angelo Paci, Abir Al Ghuzlan,
Daniel Birnbaum, Eric Baudin, Michaela Luconi, Martin Fassnacht, Bonald C. Figueiredo, François Bertucci and Enzo Lalli


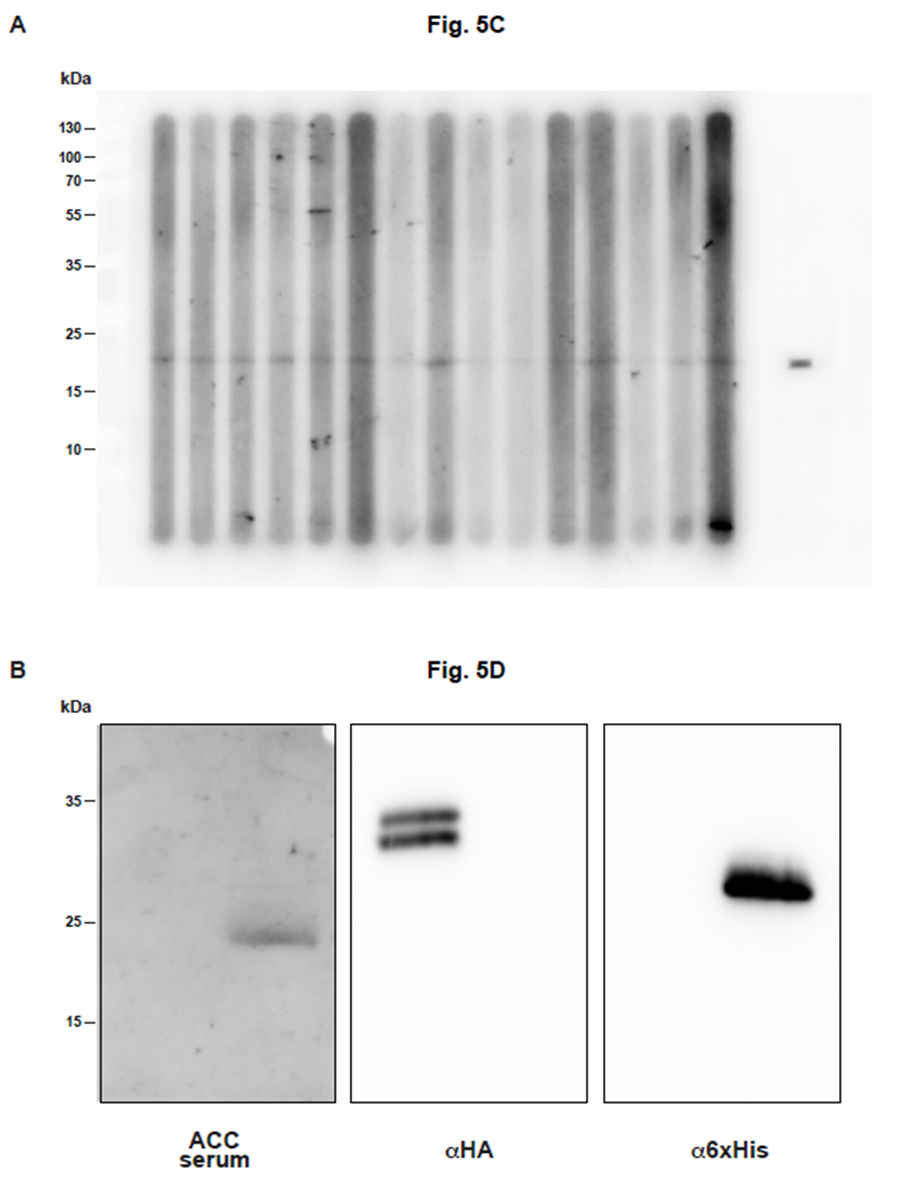


**Figure S1.** Original uncropped blots shown in Figure 5C (**A**) and Figure 5D (**B**).

| 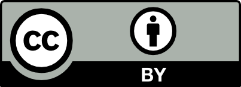 | © 2020 by the authors. Licensee MDPI, Basel, Switzerland. This article is an open access article distributed under the terms and conditions of the Creative Commons Attribution (CC BY) license (http://creativecommons.org/licenses/by/4.0/). |
| --- | --- |
